# Supplementary material for: Elevated albumin-bilirubin score as a predictor of kidney stones in adults with type 2 diabetes mellitus: evidence from a cross-sectional study
Source: PeerJ. 2025 May 14;13:e19419. doi: 10.7717/peerj.19419 (PMC12085120; doi:10.7717/peerj.19419)
Supplement: Supplemental Information 2 [file peerj-13-19419-s002.doc]

STROBE Statement—checklist of items that should be included in reports of observational studies

|  | Item No | Recommendation |
| --- | --- | --- |
| **Title and abstract** | 1 | Page 2, line 46-63 (*a*) Indicate the study’s design with a commonly used term in the title or the abstract |
|  |  | Page 2, line 49-53 (*b*) Provide in the abstract an informative and balanced summary of what was done and what was found |
| Introduction | | |
| Background/rationale | 2 | Page 2, line 67-89 Explain the scientific background and rationale for the investigation being reported |
| Objectives | 3 | Page 2, line 87-89 State specific objectives, including any prespecified hypotheses |
| Methods | | |
| Study design | 4 | Page 3, line 92-104Present key elements of study design early in the paper |
| Setting | 5 | Page 3, line 92-104Describe the setting, locations, and relevant dates, including periods of recruitment, exposure, follow-up, and data collection |
| Participants | 6 | (*a*) *Cohort study*—Give the eligibility criteria, and the sources and methods of selection of participants. Describe methods of follow-up  *Case-control study*—Give the eligibility criteria, and the sources and methods of case ascertainment and control selection. Give the rationale for the choice of cases and controls  Page 3, line 92-104 *Cross-sectional study*—Give the eligibility criteria, and the sources and methods of selection of participants |
|  |  | (*b*) *Cohort study*—For matched studies, give matching criteria and number of exposed and unexposed  *Case-control study*—For matched studies, give matching criteria and the number of controls per case |
| Variables | 7 | Page 4, line 136-155 Clearly define all outcomes, exposures, predictors, potential confounders, and effect modifiers. Give diagnostic criteria, if applicable |
| Data sources/ measurement | 8* | Page 3, line 106-134 For each variable of interest, give sources of data and details of methods of assessment (measurement). Describe comparability of assessment methods if there is more than one group |
| Bias | 9 | Page 4, line 136-155Describe any efforts to address potential sources of bias |
| Study size | 10 | Page 3, line 92-104Explain how the study size was arrived at |
| Quantitative variables | 11 | Page 4, line 136-155Explain how quantitative variables were handled in the analyses. If applicable, describe which groupings were chosen and why |
| Statistical methods | 12 | Page 4, line 157-175 (*a*) Describe all statistical methods, including those used to control for confounding |
|  |  | Page 4, line 157-175 (*b*) Describe any methods used to examine subgroups and interactions |
|  |  | Not applicable (*c*) Explain how missing data were addressed |
|  |  | (*d*) *Cohort study*—If applicable, explain how loss to follow-up was addressed  *Case-control study*—If applicable, explain how matching of cases and controls was addressed  Page 4, line 157-175*Cross-sectional study*—If applicable, describe analytical methods taking account of sampling strategy |
|  |  | Not applicable (*e*) Describe any sensitivity analyses |

Continued on next page

| Results | | |
| --- | --- | --- |
| Participants | 13* | Page 5, line 178-186 (a) Report numbers of individuals at each stage of study—eg numbers potentially eligible, examined for eligibility, confirmed eligible, included in the study, completing follow-up, and analysed |
|  |  | Page 5, line 178-186 (b) Give reasons for non-participation at each stage |
|  |  | Page 5, line 178-186 (c) Consider use of a flow diagram |
| Descriptive data | 14* | Page 5, line 178-186 (a) Give characteristics of study participants (eg demographic, clinical, social) and information on exposures and potential confounders |
|  |  | Not applicable (b) Indicate number of participants with missing data for each variable of interest |
|  |  | Not applicable (c) *Cohort study*—Summarise follow-up time (eg, average and total amount) |
| Outcome data | 15* | *Cohort study*—Report numbers of outcome events or summary measures over time |
|  |  | *Case-control study—*Report numbers in each exposure category, or summary measures of exposure |
|  |  | Page 5, line 178-186*Cross-sectional study—*Report numbers of outcome events or summary measures |
| Main results | 16 | Page 5, line 188-216 (*a*) Give unadjusted estimates and, if applicable, confounder-adjusted estimates and their precision (eg, 95% confidence interval). Make clear which confounders were adjusted for and why they were included |
|  |  | Page 5, line 188-216 (*b*) Report category boundaries when continuous variables were categorized |
|  |  | Not applicable (*c*) If relevant, consider translating estimates of relative risk into absolute risk for a meaningful time period |
| Other analyses | 17 | Page 5-6, line 218-228Report other analyses done—eg analyses of subgroups and interactions, and sensitivity analyses |
| Discussion | | |
| Key results | 18 | Page 6, line 249-260Summarise key results with reference to study objectives |
| Limitations | 19 | Page 7, line 277-288Discuss limitations of the study, taking into account sources of potential bias or imprecision. Discuss both direction and magnitude of any potential bias |
| Interpretation | 20 | Page 6-7, line 231-288Give a cautious overall interpretation of results considering objectives, limitations, multiplicity of analyses, results from similar studies, and other relevant evidence |
| Generalisability | 21 | Page 7, line 277-288Discuss the generalisability (external validity) of the study results |
| Other information | | |
| Funding | 22 | Page 1, line 16-18Give the source of funding and the role of the funders for the present study and, if applicable, for the original study on which the present article is based |

*Give information separately for cases and controls in case-control studies and, if applicable, for exposed and unexposed groups in cohort and cross-sectional studies.

**Note:** An Explanation and Elaboration article discusses each checklist item and gives methodological background and published examples of transparent reporting. The STROBE checklist is best used in conjunction with this article (freely available on the Web sites of PLoS Medicine at http://www.plosmedicine.org/, Annals of Internal Medicine at http://www.annals.org/, and Epidemiology at http://www.epidem.com/). Information on the STROBE Initiative is available at www.strobe-statement.org.
